# Prospective study on severe malaria among in-patients at Bombo regional hospital, Tanga, north-eastern Tanzania

**DOI:** 10.1186/1471-2334-11-256

**Published:** 2011-09-29

**Authors:** Hamisi A Msangeni, Mathias L Kamugisha, Samuel H Sembuche, Ezekiel K Malecela, Juma A Akida, Filbert F Temba, Bruno P Mmbando, Martha M Lemnge

**Affiliations:** 1 National Institute for Medical Research, Tanga Medical Research Centre, P.O. Box 5004, Tanga, Tanzania

## Abstract

**Background:**

In Tanzania, malaria is the major cause of morbidity and mortality, accounting for about 30% of all hospital admissions and around 15% of all hospital deaths. Severe anaemia and cerebral malaria are the two main causes of death due to malaria in Tanga, Tanzania.

**Methods:**

This was a prospective observational hospital-based study conducted from October 2004 to September 2005. Consent was sought from study participants or guardians in the wards. Finger prick blood was collected from each individual for thick and thin smears, blood sugar levels and haemoglobin estimations by Haemocue machine after admission.

**Results:**

A total of 494 patients were clinically diagnosed and admitted as cases of severe malaria. Majority of them (55.3%) were children below the age of 5 years. Only 285 out of the total 494 (57.7%) patients had positive blood smears for malaria parasites. Adults aged 20 years and above had the highest rate of cases with fever and blood smear negative for malaria parasites. Commonest clinical manifestations of severe malaria were cerebral malaria (47.3%) and severe anaemia (14.6%), particularly in the under-fives. Case fatality was 3.2% and majority of the deaths occurred in the under-fives and adults aged 20 years and above with negative blood smears.

**Conclusion:**

Proper laboratory diagnosis is crucial for case management and reliable data collection. The non-specific nature of malaria symptomatologies limits the use of clinical diagnosis and the IMCI strategy. Strengthening of laboratory investigations to guide case management is recommended.

## Background

Malaria is the leading cause of morbidity and mortality affecting about 300-400 million people worldwide and accounts for about 1-2 million deaths every year. It is also the most prevalent tropical disease especially in sub-Saharan Africa [1,2]. Major causes of malaria related deaths are severe anaemia and cerebral malaria [3-8]. According to Annual report from Bombo regional hospital for the year 2000, clinically diagnosed malaria accounted for 59.9% of all admissions of which 21.6% had had the severe form of the disease. Anaemia contributed 18.6% of all admissions. There were 228 deaths reported in the paediatric ward of which 33.3% were due to anaemia that was most likely caused by malaria. Causes of severe malaria other than anaemia accounted for 30.7% of all reported deaths [9].

Due to their low acquired immune status, children below five years of age are the most affected by malaria in high transmission areas, whereas in low transmission areas all age groups are equally affected [10,11]. Although health facility-based reports show that malaria is the leading cause for hospital attendance and admissions, several studies across Africa indicate that the disease is declining [12-14]. This has also been found in two recently conducted studies at one health facility in the city of Tanga (Makorora Health Centre), where only 10% of patients screened in one study were positive for malaria parasites [15], and in the other study (Msangeni *et al*, unpublished) data collection activities had to be stopped after failure to obtain suitable candidates despite most of the patients having clinical symptoms suggestive of malaria.

Apart from these two studies which have shown very low malaria prevalence, there is still inadequate information on the epidemiology of laboratory confirmed malaria and the underlying associated risk factors. It was the objective of this study, therefore, to determine the malaria disease pattern and the proportion of malaria associated deaths in patients admitted at Bombo regional hospital. The study was conducted for a period of one year from October 2004 to September 2005 and involved prospective data collection from severe malaria cases admitted to Bombo hospital.

## Methods

### Study area

The study was conducted in the city of Tanga, at Bombo regional hospital, north-eastern Tanzania. The city lies at about 5.17°, 5.33°S and 38.17°, 38.33°E along the Indian Ocean seashore. The area receives two seasons of rainfall; short rains during the months of October - December and long rains in March - June, with a humidity of about 100%. Temperature ranges between 27°C and 32°C. Tanga city covers an area of about 600 sq. km and has an estimated human population of 248,696 (2003 estimated at a growth rate of 2.1% per annum). The hospital has a capacity of 500 beds for inpatients among which 60 beds are reserved for paediatric patients. It has a general laboratory for both in-patient and out-patient services.

Based on data from Bombo regional hospital for the year 2003, malaria was indicated as the leading cause of hospital admissions in the intensive care unit (ICU), medical and paediatric wards, accounting for 56.8%, 39.0% and 47.5% respectively. Severe anaemia and severe malaria were noted to be the leading cause of deaths in the paediatric ICU with rates of 61.5% and 45.8% respectively. Anaemia was most probably related to malaria, especially in children.

### Study design

This was a prospective observational hospital based study conducted from 2004 to September 2005. Malaria case admissions and outcome were collected and analysed. The data were collected during official working hours and only on five working days of the week.

### Ethical consideration

Study aims were explained to Bombo hospital management team in order to make them aware and get fully involved in the study execution. Permission to conduct the study in Bombo regional hospital was sought from the Regional and City administrative authorities. During all these meetings, study aims and activities were elaborated and clarified when necessary. Oral and written consents were sought from the study participants or guardians/caretakers in the wards. Consent for adult patients with cerebral involvement was sought through relatives/caretakers. Patients' data were taken as confidential entities. The study was granted ethical clearance by the Medical Research Co-ordinating Committee of the National Institute for Medical Research.

### Clinical and laboratory data collection

Patients were admitted at the out-patient department (OPD), usually by clinical officers. The study group comprised of all patients admitted with a clinical diagnosis of severe malaria. Both sexes and all ages were eligible for inclusion in the study. Proposed diagnoses by the hospital staff were entered in the morbidity form as they were. Morbidity questionnaire was completed for all malaria admissions in the wards. Clinical reassessment and laboratory investigations were done in the ward by the research team. From each individual, personal data including name, sex, age, ward, village/street, as well as clinical and parasitological examination results were entered in the morbidity form. Clinical notes of every patient were reviewed on discharge to see if there was any re-evaluation and subsequent change of the initial diagnosis. Patients who had a change of diagnosis from malaria to another febrile illness on discharge were excluded from the study. Cerebral involvement was assessed using Blantyre coma score [16,17] by investigators.

Finger prick blood was collected from each individual for thick and thin smears. For all cases, finger prick blood was also taken for Hb level and blood sugar examinations using Haemocue machine. The blood smears were stained using 10% Giemsa for 30 minutes and examined under the microscope for identification and enumeration of malaria parasites. Malaria parasites were counted against 200 leukocytes. A blood smear was considered negative if no malaria parasites were seen after scanning 200 high power fields. When sexual parasites were present, they were enumerated against 500 leukocytes. Hyperparasitaemia was defined as parasite density ≥ 250,000 rings/μL of blood [18]. Anaemia was defined as Hb < 11 g/dL [19,20]. Severe anaemia was classified as Hb < 5 g/dL [21] whereas Hb between 5 g/dL and Hb < 11 g/dL was termed as mild/moderate anaemia [8,22]. Hypoglycaemia was defined as blood glucose < 2.2 mmole/L or < 40 mg/dL [18,23]. Fever was defined as body temperature ≥37.5°C and hyperpyrexia as temperature ≥ 39.5°C [18]. Severe malaria was reassessed according to WHO 2000 guidelines [18]. In adults, splenomegaly was defined as palpable spleen of any size but for children we considered splenomegaly when the size of the spleen exceeded 2 cm below the left costal margin. Spleen enlargement was scored according to Hackett's classification. Deaths which occurred during data collection period were also recorded.

### Treatment of severe cases in the hospital

Initial assessment was done at the OPD on admission to ascertain the presence of any severe signs which would require special attention. Parenteral/oral paracetamol was prescribed as antipyretic and analgesic whereas parenteral diazepam was prescribed to patients presenting with convulsions. Blood transfusion was provided in the case of severe anaemia (Hb < 5 g/dL) after screening for HIV, grouping and cross-matching of the blood. According to national policy quinine is the drug of choice. The drug is administered by loading and maintenance doses. There is a slight difference between the regimen for adults and for children. The loading dose for adults is 20 mg/kg body weight of quinine hydrochloride salt diluted in 10 ml/kg of 5% dextrose by intravenous infusion. The drip is left to run for over 4 hours. A maintenance dose of quinine follows 8 hours from the beginning of the previous (loading) dose and is given at 10 mg/kg in similar IV drip, again running over 4 hours. The maintenance dose is repeated every 8 hours, calculated from the beginning of the previous infusion, until patient improves to an extent that he can swallow. Then oral quinine (tablets), 10 mg salt/kg, is given 8 hourly intervals to complete a 7 days treatment course. As for children, a loading dose of quinine is administered exactly like in adults (20 mg/kg), running over 4 hours, followed by a maintenance dose (10 mg/kg) 12 hours after the start of the loading dose, given over 2 hours. This maintenance dose is repeated every 12 hours, estimated from the beginning of the previous drip. When the patient improves and can swallow, the quinine dose is given orally, 10 mg/kg 8 hourly to complete a 7 day treatment course.

### Data management and analysis

Data collected from this study were handled using Epi-info software and were double entered to minimize errors during data entry process while check files were created to restrict entrance of invalid data. Data analysis was done using STATA version 9. Proportions were compared by chisq test. Logistic regression analysis was used to compare malaria clinical signs and symptoms with blood smear results in patients admitted for severe malaria. P-value < 0.05 was considered significant.

## Results

### Patient characteristics and malaria

A total of 494 patients were clinically diagnosed and admitted as cases of severe malaria during the one year period. Males accounted for 61.5% (304) of the patients. Among the patients admitted to IPD, children aged 1-4 years were majority (34.4%), while children in the age group 5-9 years were the least (13.6%), Table 1. Most of the patients 77.5% (383) came from within the urban settings of Tanga city. A total of 245 (49.2%) patients from both rural and urban settings reported to have taken Sulphadoxine/pyrimethamine or Amodiaquine prior to admission (Table 1).

**Table 1:** Demographic characteristics of patients initially admitted with a diagnosis of severe malaria

		Age groups				
Variable	Overall	Infants	1-4 years	5-9 years	10-19 years	20+ years
Number enrolled	494	103	170	36	67	118
Sex ratio (M/F)	1.6:1	2:01	1:01	1:02	3.5:1	2.4:1
Mean age (Years, SD)	12.2	0.6	2.5	6.9	15.2	36.1
Mean axillary temp (°C) [Range]	38.5 (36.0-41.2)	38.4 (36.5-40.6)	38.5 (36.0-41.2)	38.3 (36.3-40.5)	38.2 (36.0-41.0)	38.2 (36.0-40.5)
Malaria parasite density (GMPD)/μL	5420	5082	6397	2728	10734	2158
GMPD^†† ^(95% CI)	(2472-13981)	(2583-10599)	(4027-10428)	(880-10207)	(3912-33307)	(956-5363)
Mean Hb (g/dl, range)	8.9 (1.6-17.6)	7.7 (2.2-15.4)	7.4 (1.6-15.6)	9.1 (4.0-14.3)	11.4 (4.3-17.6)	10.8 (2.9-16.9)
Malaria parasite prevalence (%)	57.7	58 (20.3)	121 (42.5)	27 (9.5)	39 (13.7)	40 (14.0)
Fever (axillary temp ≥ 37.5°C) %	348 (70.5)	78 (22.4)	130 (37.4)	26 (7.5)	40 (11.5)	74 (21.2)
Severe anaemia* (%)	72 (14.6)	21 (29.2)	37 (51.4)	3 (4.2)	5 (6.9)	6 (8.3)
Mild/moderate anaemia† (%)	273 (55.3)	64 (23.4)	114 (41.8)	24 (8.8)	24 (8.8)	47 (17.2)
Splenomegaly prevalence (%)	145 (29.3)	39 (26.9)	62 (42.8)	9 (6.2)	18 (12.4)	17 (11.7)

* Severe anaemia (Hb < 5 g/dL)

† Mild/moderate anaemia (Hb > 5 g/dl - Hb < 11 g/dL)

^†† ^GMPD = Geometric mean parasite density among observed positive cases only.

Overall, 57.7% (285) of the cases had positive blood smears for malaria parasites. *Plasmodium falciparum *and *P. malariae *were the only malaria parasites species detected. *P. malariae *was only found as mixed infection and it accounted for 6.7% (19) of all infections. Highest smear positive rate was recorded in children under the age of 5 years and thereafter, the prevalence showed a sharp drop that levelled off after the age of 10 years (Table 1).

The highest parasite density of 10734 asexual parasites/μL (95% CI: 3912 - 33307) was recorded in patients aged 10-19 years whilst the lowest parasite density (2158 asexual parasites/μL, 95% CI: 956 - 5363) was seen in adults aged ≥20 years, (Table 1).

### Fever and malaria parasites

**Figure 1:**
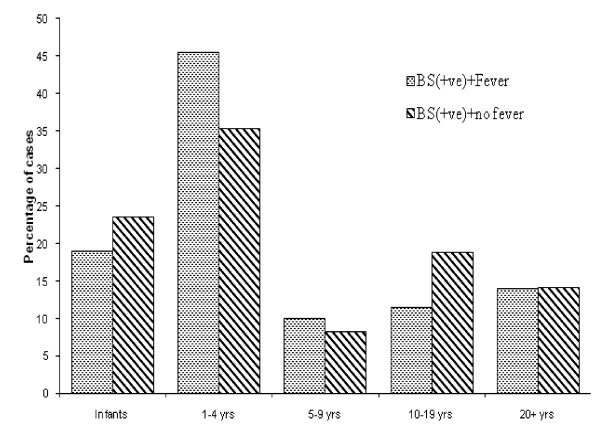
Comparison of blood smear with fever episodes in cases admitted for severe malaria by age groups

About 70% of the patients admitted with a diagnosis of severe malaria had fever (axillary temperature ≥ 37.5°C). The highest rate of fever was recorded in children aged 1-4 years followed by infants and adults aged ≥20 years. Figure 1 shows results of fever in association with blood smears. Children in age group 1-4 years had the highest fever prevalence in association with blood smear (BS) positive for malaria parasites (Figure 1). Adults aged ≥20 years had a high proportion of negative BS with no fever whereas children aged 5-9 years had the lowest rate of both fever and malaria parasites.

### Anaemia

Anaemia was a prominent feature among the patients, with an overall rate of 69.8% (345). Only 30.2% (149/494) of the patients had normal Hb levels (Hb > 11 g/dL). Majority (55.2%) of the cases had mild/moderate anaemia. Severe anaemia was recorded in 14.6% (72) of the patients and a significant proportion of them *(*84.7%, *p = 0.001) *had positive BS for malaria parasites. The prevalence of severe anaemia was highest in children under the age of 5 years and decreased with increasing age.

### Severe malaria manifestations

Following clinical reassessment of patients in the ward, the proportion of patients with a clinical confirmed diagnosis of severe malaria was 74.7% (369), (Table 2). Out of 369 cases, a significant proportion (61.5%) had positive BS for malaria parasites *(p = 0.003)*. Most of these patients were children below the age of 5 years. Assessment by the Blantyre coma scale revealed that 129 out of total 273 under-fives (47.3%) admitted had signs and symptoms indicative of cerebral malaria but only 89 of the 129 children (69.0%) had positive BS for malaria parasites (Table 2).

**Table 2:** Comparison of malaria clinical signs and symptoms with blood smear results in patients admitted for severe malaria at Bombo hospital, Tanga, Tanzania

	Clinical observation alone					
					
Signs & symptoms	Initial total cases*+	Reassessed cases (n,%)**+	No. +ve (n,%)	Odds ratio	*P-value*	95% CI
Clinical manifestations*	494	369 (74.7)	227 (61.5)	1.63	0.008	1.136 - 2.350
Cerebral malaria**	273	129 (47.3)	89 (69.0)	1.34	0.26	0.807 - 2.208
Severe anaemia	494	72 (14.6)	61 (84.7)	4.85	0.001	2.509 - 9.578
Hyperpyrexia†	494	97 (19.6)	59 (60.8)	1.17	0.486	0.746 - 1.849
Hyperparasitaemia††	494	285 (57.7)	4 (1.4)		0.085	
Convulsions	494	23 (4.7)	17 (73.9)	2.15	0.107	0.831 - 5.540
Jaundice	494	6 (1.2)	3 (50.0)	0.73	0.701	0.146 - 3.656

*+Initial total cases are those admitted with diagnosis of severe malaria from OPD

**+Reassessed cases are those confirmed to have severe malaria by investigators.

*Clinical signs and symptoms used as inclusion criteria for severe malaria

**According to the Blantyre coma score scale

†Axillary temperature ≥ 39.5°C

††Parasite count ≥ 250,000/μL of blood

Hyperpyrexia, hyperparasitaemia, hypoglycaemia, convulsions and jaundice were among the accompanying clinical complications of severe malaria observed across all age groups. Among all these complications, hyperpyrexia appeared to be relatively high (19.6%) (Table 2). Again, the proportion of patients having any of these conditions with malaria parasitaemia was not significant. Fever was identified in 70.5% (348) of cases but the proportion of those with positive BS for malaria parasites was significantly higher *(p = 0.006) *only in infants (Table 2 & 3). Hyperpyrexia was most prominent in those with positive BS with the exception of infants and the adult age groups who showed comparatively higher proportions of hyperpyrexia in those with negative BS. Hypoglycaemia was not a major clinical problem as it was seen only in 5 out of the 494 (1%) patients of whom only one had positive BS for malaria parasites. Only 4 out of the 494 (0.8%) patients recruited had hyperparasitaemia, and all of them were children under the age of five years. There was a significantly higher proportion (85.5%) of children aged 1-4 with both malaria and enlarged spleen (*p = 0.001*) as shown in Table 3.

**Table 3:** Relationship between clinical conditions and presence of malaria parasites by age group

		Age group				
Signs/Symptoms	Status	Infants	1-4 yrs	5-9 yrs	10-19 yrs	20+ yrs
	No	3 (20.0)	4 (33.3)	4 (50.0)	21 (56.8)	26 (49.1)
Severe clinical manifestations (%)	Yes	12 (62.5)	117 (74.1)	23 (82.1)	18 (60.0)	14 (21.5)
	*P- value*	0.002	0.003	0.064	0.789	0.002
	No	20 (80.0)	30 (75.0)	7 (70.0)	16 (59.3)	12 (27.3)
Fever (Temp ≥ 37.5°, %)	Yes	38 (48.7)	91 (70.0)	20 (76.9)	23 (57.5)	28 (37.8)
	*P- value*	0.006	0.542	0.667	0.886	0.241
	No	4 (58.6)	49 (66.2)			
Cerebral malaria* (%)	Yes	17 (51.5)	72 (75.0)			
	*P- value*	0.5	0.21			
	No	38 (46.3)	86 (64.7)	25 (75.8)	37 (59.7)	38 (33.9)
Severe anaemia (Hb < 5 g/dl, %)	Yes	20 (95.2)	35 (94.6)	2 (66.7)	2 (40.0)	2 (33.3)
	*P- value*	0.0001	0.0001	0.728	0.391	0.976
	No	53 (55.8)	112 (72.3)	27 (75.0)	37 (56.9)	38 (33.0)
Convulsions (%)	Yes	4 (66.7)	9 (69.2)	0	2 (100)	2 (100)
	*P- value*	0.602	0.815		0.224	0.048
	No	28 (43.8)	68 (63.0)	20 (74.1)	31 (63.3)	35 (34.7)
Splenomegaly prevalence (%)	Yes	30 (76.9)	53 (85.5)	7 (77.8)	8 (44.4)	5 (29.4)
	*P- value*	0.001	0.002	0.824	0.166	0.673

*Assessed by the Blantyre coma score scale

### Duration of illness

The majority of patients in the study had been sick for varying lengths before admission, ranging from 1 to 90 days. The longest period of sickness was documented in adults of 20 years and above who had a median duration of sickness of 7 [IQ range: 4-33] days. On the other hand, a high proportion of the adults aged 20 years and above gave a history of being sick for durations exceeding 2 weeks (Table 4). Generally, the proportion of days of illness seemed to increase with advancing age. Majority of the patients who had positive BS for malaria parasites on admission claimed to have been sick for periods not exceeding one week except a few in the age group of 1-4 years, 10-19 years and adults of 20 years and above. Patients who were admitted with negative BS for malaria parasites reported the longest periods of sickness, the mean duration increasing progressively from 5.6 [95% CI 4.2 - 7.0] days in infants to 21.8 [95% CI 15 - 28.2] days in adults aged 20 years and above (Table 4).

**Table 4:** Comparison of duration of illness with blood smear results for malaria parasites

Variable	Age group					
**Duration of illness (days) with BS positive**	**Infants**	**1-4 years**	**5-9 years**	**10-19 years**	**20+ years**	**Total**

	(%)	(%)	(%)	(%)	(%)	
≤ 7 days	53 (20.6)	109 (42.4)	24 (9.3)	35 (13.6)	36 (14.0)	257
8-14 days	5 (25.0)	8 (40.0)	3 (15.0)	1 (5.0)	3 (15.0)	20
15+ days	0	4 (50.0)	0	3 (37.5)	1 (12.5)	8
**Median duration (days) of illness [IQ range]**	**4**[3-6]	**4 **[3,6]	**6 **[3,6]	**4 **[3-6]	**4 **[3-6]	

**Duration of illness (days) with BS negative**	**Infants**	**1-4 years**	**5-9 years**	**10-19 years**	**20+ years**	**Total**

	(%)	(%)	(%)	(%)	(%)	
≤ 7 days	39 (25.2)	42 (27.1)	7 (4.5)	25 (16.1)	42 (27.1)	155
8-14 days	5 (22.7)	5 (22.7)	2 (9.1)	1 (4.6)	9 (40.9)	22
15+ days	1 (3.0)	2 (6.3)	0	2 (6.3)	27 (84.4)	33
**Median duration (days) of illness [IQ range]**	**4[3- 7.0]**	**4 **[3-7]	**5 **[5-7]	**4.5 **[3-7]	**7 **[4-33]	

### Deaths

Most of the patients (95.8%) were successfully treated and discharged. However, 5 (1.0%) were transferred to other health facilities for various reasons and 16 (3.2%) of the 494 patients died during the one year period. Most of the deaths occurred in patients from the urban areas of Tanga city (81.2% vs 18.3%). Deaths reported were of infants (12.5%), children aged 1-4 years (43.8%) and adults (37.5%) of over 19 years of age, and all of the deceased adults had negative BS for malaria parasites. Only 6 out of these 16 (37.5%) deaths had positive BS for malaria parasites of whom 2 were infants and 4 were children aged 1-4 years.

## Discussion

According to health facility statistics, malaria in Tanzania is the leading cause of morbidity and mortality. Ministry of Health data from 2003 show that around 30 per cent of all hospital admissions and 15 per cent of all hospital deaths in Tanzania are due to malaria [24]. Results obtained in our study show that just over half of the patients admitted in the 1 year period October 2004 to September 2005 were cases of malaria. A case of malaria requires the presence of parasites in the blood, supported by fever or history of fever. Reyburn [25] recorded almost similar findings in a study aimed at assessing the diagnosis and outcomes in patients admitted with a diagnosis of severe malaria in north-eastern Tanzania. In the case of fever in BS negative, infections other than malaria should be considered as has been documented by Berkley [26] in Kenyan children where various bacterial infections were found to be responsible for the majority of childhood deaths.

An interesting observation was that a considerable proportion of patients with negative blood smear for malaria parasites and without fever was also admitted as severe cases of malaria. The inclusion of afebrile/apyrexic patients and those with BS negative results for malaria parasites as severe malaria shows that malaria is over-reported in this health facility using clinical diagnosis as the principal method of diagnosis. This is likely to occur in most Sub-Saharan Africa and is facilitated through the syndromic approach, popularly termed as integrated management of childhood illness (IMCI) strategy [27], which is not a precise way of diagnosing diseases [28,29]. Reyburn [25] had comparable findings where only 46.1% of the clinically examined patients had positive blood smears.

The high rate of cases with negative BS for malaria parasites found in our study could have been attributed to the inclusion of patients with acute febrile illnesses caused by aetiologies other than malaria. This is similar to what Njama-Meya [30] reported in a prospective cohort study of malaria treatment restricted to laboratory-confirmed cases in Ugandan children in which 32% of the febrile episodes were found positive for malaria. This means that majority (68%) of the episodes attended were caused by various other infections.

The proportion of cases with hypoglycaemia in our study was relatively lower than those reported in other areas of Sub-Sahara Africa. For instance, Taylor *et al *[31] in a study to find out the clinical associations and relationship to quinine dosage of hypoglycaemia in severe malaria in Malawi reported that 20% of children had hypoglycaemia before treatment. In another study that was conducted along the coastal areas of Kenya, English *et al *[32] reported almost similar proportion (16%) of hypoglycaemia in children on admission. So far, we cannot identify the reasons for the lower proportion of hypoglycaemia recorded in patients in our study.

Moreover, the low sensitivity of laboratory techniques used in diagnosing malaria could have played a part in the increase of BS negative results. Microscopy has some technical drawbacks that make it less sensitive than polymerase chain reaction (PCR) technique in the detection of malaria parasites [33]. It has been noted that considerable number of parasites are lost during the staining process [34-36], leading to either incorrect estimates of parasite density or the smear being considered as negative in the case of scanty or low parasitaemia, usually in densities of < 50 parasites/μL of blood [37,38]. Otherwise these children had infections in other body systems causing illnesses resembling severe malaria, e.g. septicaemia, pneumonia, urinary tract infection, HIV/AIDS and other viral infections.

According to the scores obtained with the Blantyre coma scale, it seems that cerebral malaria is a common manifestation of severe malaria among the under-fives in this area. It was observed that among the sick children assessed, 47.3% had altered consciousness indicative of cerebral involvement, and about one third of these had negative BS for malaria parasites. Considering the fact that a case definition of malaria requires the presence of asexual parasites in the blood[39,40], it is most likely that the patients who had no parasitaemia could have other infections with cerebral manifestations mimicking cerebral malaria, like cerebrospinal meningitis [41]. The proportion of patients who had cerebral malaria (47.3%) was actually higher than that recorded by Pankoui Mfonkeu (20.2%) in Douala, Cameroon [42].

The considerable proportion of patients observed with signs and symptoms of severe malaria, and others presenting with hyperpyrexia (body temperature ≥ 39.5°C) but having no parasitaemia indicates very serious diagnostic errors and case management problems in our hospitals. In this study, for instance, 38.5% of patients who presented with severe clinical signs and symptoms of disease pertaining to severe malaria and 19.6% of patients who had hyperpyrexia had no parasitaemia. This in part is due to inadequate quality of care resulting from dependence on under-trained personnel for most of the clinical work in most of our health facilities, and absence of or insufficient laboratory diagnostic services, particularly in the primary health care units (Mangesho *et al*. in preparation). Unfortunately under-trained personnel are normally the first to be consulted even in most regional hospitals in Tanzania. It is true that malaria is the leading cause of hospital attendances and hospital admissions in Tanzania so far and probably in sub-Saharan Africa as a whole. But the findings of this study portray doubt on the reliability of the accuracy of data from health facilities.

Furthermore, severe anaemia also appeared to be a problem that was significantly associated with malaria (p = 0.001). The trend showed that where there was an increase in the proportion of BS positive results for malaria parasites, there was a corresponding increase in the proportion of severe anaemia cases, and vice versa. This phenomenon was more marked in children below the age of five years who also happened to be the group with the highest proportion of BS positive for malaria parasites. In many malaria endemic countries anaemia has been reported to be the most common complication of falciparum malaria and one of the commonest causes of deaths in children [5,43,44]. The overall prevalence of malaria-related severe anaemia in our study was 14.6%, which was almost threefold less compared to 46.0% reported by Newton [45] in children along the coastal area of Kenya between 1989 and 1991 and, by Dzeing-Ella [21] in Gabon between 2000 and 2002, where prevalence of severe anaemia has been reported to be as high as 67.%. In general, the prevalence of malaria-related severe anaemia has a corresponding level with malaria endemicity, for instance, Font [46] found that malaria and anaemia were the main causes of referrals from rural health facilities to the district hospital of Kilombero in Tanzania. Effective malaria control interventions, particularly the country-wide use of insecticide treated bed nets [47,48] might have been responsible for the decline in malaria prevalence recently reported in Tanzania [49], resulting in a parallel decrease in the level of severe anaemia recorded in our study. However, despite the possible association between malaria and anaemia, it is difficult to draw a strong conclusion of malaria being the sole cause and effect of anaemia due to its multi-factorial nature in the tropical environment [50].

It has been noted that patients reported various duration of illness. Some had been sick for periods as short as one day, whereas a number of them were unwell for almost 3 months. Duration of illness documented in adult patients (≥20 years) was noted to be significantly long among those with negative BS, indicating that some causes other than malaria, like HIV/AIDS, might have been responsible for the sickness in this age group.

Low death rates might have resulted from good access to health services for the majority of the patients living within the city (as has been evidenced by the fact that half of the patients already received some antimalarials even before coming to hospital). Prompt treatment by hospital staff due in part to over-diagnosis of malaria [25] played a role in the low rate of malaria-specific deaths. The use of quinine has proved to be very effective in reducing mortality in our study although recent report indicates that artesunate performs better [51].

Almost half of the patients enrolled in our study had a history of taking antimalarial drugs prior to admission. These patients most likely got their medicines either from low level health facilities before they were referred or they might have obtained the drugs over the counter, which is a common practice in Tanzania. Some studies conducted in the country show that over 71% of patients treat themselves even before consulting a health facility [52,53].

## Conclusions

The high proportion of fever cases in patients with BS negative results for malaria parasites poses the question as to what could be the actual cause of illness. There is a need to find out if malaria still continues to be the leading cause of morbidity and mortality in Tanzania and sub-Saharan Africa as is believed to be, particularly bearing in mind the impact of HIV/AIDS. Use of more specific RDT might also help. This would reduce the problem of over-diagnosis of malaria and bring about rational use of antimalarials and other relatively expensive drugs. We need to revisit the usefulness of IMCI in terms of its impact on disease over-diagnosis and over-prescription of drugs. Improvement of clinical and laboratory diagnosis of malaria is crucial for proper case management and reliable data collection in the changing epidemiological setting. Blood and urine culture tests could be an important component of management of febrile illnesses in general and cases of non malaria febrile illness in particular.

## Competing interests

The authors declare that they have no competing interests.

## Authors' contributions

HAM designed the study, collected data and prepared the manuscript. MLK designed the study, analyzed the data and prepared the manuscript. MML designed the study and prepared the manuscript. BPM and FFT participated in data analysis. SHS, EKM and JAA collected data. All authors read and approved the final manuscript.

## Pre-publication history

The pre-publication history for this paper can be accessed here:

http://www.biomedcentral.com/1471-2334/11/256/prepub

## References

[B1] WHO The African Malaria Report 2003

[B2] ter Kuile FO Parise ME Verhoeff FH Udhayakumar V Newman RD van Eijk AM Rogerson SJ Steketee RW The burden of co-infection with human immunodeficiency virus type 1 and malaria in pregnant women in sub-saharan Africa Am J Trop Med Hyg 2004 71 41 54 15331818

[B3] Severe falciparum malaria. World Health Organization, Communicable Diseases Cluster Trans R Soc Trop Med Hyg 2000 94 Suppl 1 S1 90 11103309

[B4] Slutsker L Taylor TE Wirima JJ Steketee RW In-hospital morbidity and mortality due to malaria-associated severe anaemia in two areas of Malawi with different patterns of malaria infection Trans R Soc Trop Med Hyg 1994 88 548 551 10.1016/0035-9203(94)90157-0 7992335

[B5] Menendez C Fleming AF Alonso PL Malaria-related anaemia Parasitol Today 2000 16 469 476 10.1016/S0169-4758(00)01774-9 11063857

[B6] Kitua AY Smith TA Alonso PL Urassa H Masanja H Kimario J Tanner M The role of low level Plasmodium falciparum parasitaemia in anaemia among infants living in an area of intense and perennial transmission Trop Med Int Health 1997 2 325 333 9171840 10.1111/j.1365-3156.1997.tb00147.x

[B7] Granja AC Machungo F Gomes A Bergstrom S Brabin B Malaria-related maternal mortality in urban Mozambique Ann Trop Med Parasitol 1998 92 257 263 10.1080/00034989859816 9713540

[B8] Marsh K Forster D Waruiru C Mwangi I Winstanley M Marsh V Newton C Winstanley P Warn P Peshu N Indicators of life-threatening malaria in African children N Engl J Med 1995 332 1399 1404 10.1056/NEJM199505253322102 7723795

[B9] The United Republic of Tanzania Prime Minister's Office Regional Administration and Local Government: Annual Report Bombo Hospital-Tanga 2000

[B10] Bodker R Akida J Shayo D Kisinza W Msangeni HA Pedersen EM Lindsay SW Relationship between altitude and intensity of malaria transmission in the Usambara Mountains, Tanzania J Med Entomol 2003 40 706 717 10.1603/0022-2585-40.5.706 14596287

[B11] Mmbando BP Segeja MD Msangeni HA Sembuche SH Ishengoma DS Seth MD Francis F Rutta AS Kamugisha ML Lemnge MM Epidemiology of malaria in an area prepared for clinical trials in Korogwe, north-eastern Tanzania Malar J 2009 8 165 10.1186/1475-2875-8-165 19615093 PMC2720983

[B12] Ceesay SJ Casals-Pascual C Nwakanma DC Walther M Gomez-Escobar N Fulford AJ Takem EN Nogaro S Bojang KA Corrah T Continued decline of malaria in The Gambia with implications for elimination PLoS One 5 e12242 10.1371/journal.pone.0012242 PMC2923605 20805878

[B13] Gething PW Smith DL Patil AP Tatem AJ Snow RW Hay SI Climate change and the global malaria recession Nature 465 342 345 10.1038/nature09098 PMC2885436 20485434

[B14] Okiro EA Hay SI Gikandi PW Sharif SK Noor AM Peshu N Marsh K Snow RW The decline in paediatric malaria admissions on the coast of Kenya Malar J 2007 6 151 10.1186/1475-2875-6-151 18005422 PMC2194691

[B15] Kamugisha ML Msangeni H Beale E Malecela EK Akida J Ishengoma DR Lemnge MM Paracheck Pf compared with microscopy for diagnosis of Plasmodium falciparum malaria among children in Tanga City, north-eastern Tanzania Tanzan J Health Res 2008 10 14 19 18680960 10.4314/thrb.v10i1.14336

[B16] Molyneux ME Taylor TE Wirima JJ Borgstein A Clinical features and prognostic indicators in paediatric cerebral malaria: a study of 131 comatose Malawian children Q J Med 1989 71 441 459 2690177

[B17] Waller D Krishna S Crawley J Miller K Nosten F Chapman D ter Kuile FO Craddock C Berry C Holloway PA Clinical features and outcome of severe malaria in Gambian children Clin Infect Dis 1995 21 577 587 10.1093/clinids/21.3.577 8527547

[B18] World Health Organisation Manegement of severe malaria-A practical handbook 2rd edition 2000 Geneva: WHO

[B19] Tomashek KM Woodruff BA Gotway CA Bloland P Mbaruku G Randomized intervention study comparing several regimens for the treatment of moderate anemia among refugee children in Kigoma Region, Tanzania Am J Trop Med Hyg 2001 64 164 171 11442213 10.4269/ajtmh.2001.64.164

[B20] Odhiambo FO Hamel MJ Williamson J Lindblade K ter Kuile FO Peterson E Otieno P Kariuki S Vulule J Slutsker L Newman RD Intermittent preventive treatment in infants for the prevention of malaria in rural Western kenya: a randomized, double-blind placebo-controlled trial PLoS One 5 e10016 10.1371/journal.pone.0010016 PMC2848869 20368815

[B21] Dzeing-Ella A Nze Obiang PC Tchoua R Planche T Mboza B Mbounja M Muller-Roemer U Jarvis J Kendjo E Ngou-Milama E Severe falciparum malaria in Gabonese children: clinical and laboratory features Malar J 2005 4 1 10.1186/1475-2875-4-1 15638948 PMC546207

[B22] Idro R Severe anaemia in childhood cerebral malaria is associated with profound coma Afr Health Sci 2003 3 15 18 12789083 PMC2141586

[B23] Mockenhaupt FP Ehrhardt S Burkhardt J Bosomtwe SY Laryea S Anemana SD Otchwemah RN Cramer JP Dietz E Gellert S Bienzle U Manifestation and outcome of severe malaria in children in northern Ghana Am J Trop Med Hyg 2004 71 167 172 15306705

[B24] Ministry of Health Health Abstracts 2003 Dar es salaam, United Republic of Tanzania: Ministry of Health

[B25] Reyburn H Mbatia R Drakeley C Carneiro I Mwakasungula E Mwerinde O Saganda K Shao J Kitua A Olomi R Overdiagnosis of malaria in patients with severe febrile illness in Tanzania: a prospective study BMJ 2004 329 1212 10.1136/bmj.38251.658229.55 15542534 PMC529364

[B26] Berkley JA Lowe BS Mwangi I Williams T Bauni E Mwarumba S Ngetsa C Slack MP Njenga S Hart CA Bacteremia among children admitted to a rural hospital in Kenya N Engl J Med 2005 352 39 47 10.1056/NEJMoa040275 15635111

[B27] Font F Alonso Gonzalez M Nathan R Kimario J Lwilla F Ascaso C Tanner M Menendez C Alonso PL Diagnostic accuracy and case management of clinical malaria in the primary health services of a rural area in south-eastern Tanzania Trop Med Int Health 2001 6 423 428 10.1046/j.1365-3156.2001.00727.x 11422955

[B28] Adehor AB Burrell PR The Integrated Management of Health Care Strategies and Differential Diagnosis by Expert System Technology: A Single-Dimensional Approach 2008 http://www.waset.org/journals/waset/v44-91.pdf

[B29] Rowe AK Hirnschall G Lambrechts T Bryce J Linking the integrated management of childhood illness (IMCI) and health information system (HIS) classifications: issues and options Bull World Health Organ 1999 77 988 995 10680246 PMC2557775

[B30] Njama-Meya D Clark TD Nzarubara B Staedke S Kamya MR Dorsey G Treatment of malaria restricted to laboratory-confirmed cases: a prospective cohort study in Ugandan children Malar J 2007 6 7 10.1186/1475-2875-6-7 17239256 PMC1797179

[B31] Taylor TE Molyneux ME Wirima JJ Fletcher KA Morris K Blood glucose levels in Malawian children before and during the administration of intravenous quinine for severe falciparum malaria N Engl J Med 1988 319 1040 1047 10.1056/NEJM198810203191602 3050516

[B32] English M Wale S Binns G Mwangi I Saurewein H Marsh K Hypoglycaemia on and after admission in Kenyan children with severe malaria Q J Med 1998 91 191 197 10.1093/qjmed/91.3.191 9604071

[B33] Coleman RE Sattabongkot J Promstaporm S Maneechai N Tippayachai B Kengluecha A Rachapaew N Zollner G Miller RS Vaughan JA Comparison of PCR and microscopy for detection of asymptomatic malaria in a Plasmoium falciparum/vivax endemic area in Thailand Malar J 2006 5 10.1186/1475-2875-5-121 PMC1716172 17169142

[B34] Shute PG The staining of malaria parasites Trans R Soc Trop Med Hyg 1966 60 412 416 10.1016/0035-9203(66)90311-7 4162149

[B35] Bejon P Andrews L Hunt-Cooke A Sanderson F Gilbert SC Hill AV Thick blood film examination for Plasmodium falciparum malaria has reduced sensitivity and underestimates parasite density Malar J 2006 5 104 10.1186/1475-2875-5-104 17092336 PMC1636647

[B36] Dowling MA Shute GT A comparative study of thick and thin blood films in the diagnosis of scanty malaria parasitaemia Bull World Health Organ 1966 34 249 267 5296131 PMC2475932

[B37] Wongsrichanalai C Barcus MJ Muth S Sutamihardja A Wernsdorfer WH A review of malaria diagnostic tools: microscopy and rapid diagnostic test (RDT) Am J Trop Med Hyg 2007 77 119 127 18165483

[B38] Tangpukdee N Duangdee C Wilairatana P Krudsood S Malaria diagnosis: a brief review Korean J Parasitol 2009 47 93 102 10.3347/kjp.2009.47.2.93 19488414 PMC2688806

[B39] Redd SC Bloland PB Kazembe PN Patrick E Tembenu R Campbell CC Usefulness of clinical case-definitions in guiding therapy for African children with malaria or pneumonia Lancet 1992 340 1140 1143 10.1016/0140-6736(92)93160-O 1359219

[B40] Rogers WO Atuguba F Oduro AR Hodgson A Koram KA Clinical case definitions and malaria vaccine efficacy J Infect Dis 2006 193 467 473 10.1086/499314 16388497

[B41] Wright PW Avery WG Ardill WD McLarty JW Initial clinical assessment of the comatose patient: cerebral malaria vs. meningitis Pediatr Infect Dis J 1993 12 37 41 10.1097/00006454-199301000-00009 8417424

[B42] Mfonkeu JB Gouado I Kuate HF Zambou O Grau G Combes V Zollo PH Clinical presentation, haematological indices and management of children with severe and uncomplicated malaria in Douala, Cameroon Pak J Biol Sci 2008 11 2401 2406 10.3923/pjbs.2008.2401.2406 19137849

[B43] Rogerson S What is the relationship between haptoglobin, malaria, and anaemia? PLoS Med 2006 3 e200 10.1371/journal.pmed.0030200 16637743 PMC1450019

[B44] Ronald LA Kenny SL Klinkenberg E Akoto AO Boakye I Barnish G Donnelly MJ Malaria and anaemia among children in two communities of Kumasi, Ghana: a cross-sectional survey Malar J 2006 5 105 10.1186/1475-2875-5-105 17094806 PMC1654171

[B45] Newton CR Warn PA Winstanley PA Peshu N Snow RW Pasvol G Marsh K Severe anaemia in children living in a malaria endemic area of Kenya Trop Med Int Health 1997 2 165 178 10.1046/j.1365-3156.1997.d01-238.x 9472302

[B46] Font F Quinto L Masanja H Nathan R Ascaso C Menendez C Tanner M Schellenberg J Alonso P Paediatric referrals in rural Tanzania: the Kilombero District Study - a case series BMC Int Health Hum Rights 2002 2 4 10.1186/1472-698X-2-4 11983024 PMC111197

[B47] Bernard J Mtove G Mandike R Mtei F Maxwell C Reyburn H Equity and coverage of insecticide-treated bed nets in an area of intense transmission of Plasmodium falciparum in Tanzania Malar J 2009 8 65 10.1186/1475-2875-8-65 19371415 PMC2674468

[B48] Hanson K Marchant T Nathan R Mponda H Jones C Bruce J Mshinda H Schellenberg JA Household ownership and use of insecticide treated nets among target groups after implementation of a national voucher programme in the United Republic of Tanzania: plausibility study using three annual cross sectional household surveys BMJ 2009 339 b2434 10.1136/bmj.b2434 19574316 PMC2714691

[B49] Mmbando BP Vestergaard LS Kitua AY Lemnge MM Theander TG Lusingu JP A progressive declining in the burden of malaria in north-eastern Tanzania Malar J 9 216 10.1186/1475-2875-9-216 PMC2920289 20650014

[B50] Crawley J Reducing the burden of anemia in infants and young children in malaria-endemic countries of Africa: from evidence to action Am J Trop Med Hyg 2004 71 25 34 15331816

[B51] Dondorp AM Fanello CI Hendriksen IC Gomes E Seni A Chhaganlal KD Bojang K Olaosebikan R Anunobi N Maitland K Artesunate versus quinine in the treatment of severe falciparum malaria in African children (AQUAMAT): an open-label, randomised trial Lancet 2010 376 1647 1657 10.1016/S0140-6736(10)61924-1 21062666 PMC3033534

[B52] Nsimba SE Rimoy GH Self-medication with chloroquine in a rural district of Tanzania: a therapeutic challenge for any future malaria treatment policy change in the country J Clin Pharm Ther 2005 30 515 519 10.1111/j.1365-2710.2005.00645.x 16336283

[B53] Mnyika KS Killewo JZ Kabalimu TK Self-medication with antimalarial drugs in Dar es Salaam, Tanzania Trop Geogr Med 1995 47 32 34 7747329

